# Supplementary material for: Modeling glioblastoma heterogeneity as a dynamic network of cell states
Source: Mol Syst Biol. 2021 Sep 16;17(9):e10105. doi: 10.15252/msb.202010105 (PMC8444284; doi:10.15252/msb.202010105)
Supplement: Supplementary file 6 — Source Data for Figure 5 [file MSB-17-e10105-s004.zip › Figure5A_sourcedata/GSEA_3071/hallmarks_stateII.GseaPreranked.1623742536692/HALLMARK_MITOTIC_SPINDLE.html]

Details for gene set HALLMARK\_MITOTIC\_SPINDLE[GSEA]

|  || Dataset | state23071 |
| Phenotype | NoPhenotypeAvailable |
| Upregulated in class | na\_pos |
| GeneSet | HALLMARK\_MITOTIC\_SPINDLE |
| Enrichment Score (ES) | 0.46748212 |
| Normalized Enrichment Score (NES) | 2.8382487 |
| Nominal p-value | 0.0 |
| FDR q-value | 0.0 |
| FWER p-Value | 0.0 |
Table: GSEA Results Summary

  

Fig 1: Enrichment plot: HALLMARK\_MITOTIC\_SPINDLE      
 Profile of the Running ES Score & Positions of GeneSet Members on the Rank Ordered List

  

| PROBE | GENE SYMBOL | GENE\_TITLE | RANK IN GENE LIST | RANK METRIC SCORE | RUNNING ES | CORE ENRICHMENT || 1 | ANLN |  |  | 4 | 1.760 | 0.0283 | Yes |
| 2 | CDK1 |  |  | 14 | 1.540 | 0.0517 | Yes |
| 3 | BRCA2 |  |  | 22 | 1.445 | 0.0740 | Yes |
| 4 | FBXO5 |  |  | 30 | 1.359 | 0.0948 | Yes |
| 5 | TOP2A |  |  | 72 | 1.132 | 0.1034 | Yes |
| 6 | KIF2C |  |  | 73 | 1.132 | 0.1223 | Yes |
| 7 | ARHGAP29 |  |  | 85 | 1.103 | 0.1379 | Yes |
| 8 | NDC80 |  |  | 94 | 1.074 | 0.1538 | Yes |
| 9 | TPX2 |  |  | 111 | 1.025 | 0.1668 | Yes |
| 10 | KIF11 |  |  | 115 | 1.014 | 0.1830 | Yes |
| 11 | KIF20B |  |  | 133 | 0.989 | 0.1952 | Yes |
| 12 | KIF23 |  |  | 161 | 0.936 | 0.2040 | Yes |
| 13 | SAC3D1 |  |  | 188 | 0.887 | 0.2122 | Yes |
| 14 | NET1 |  |  | 220 | 0.857 | 0.2188 | Yes |
| 15 | RACGAP1 |  |  | 241 | 0.831 | 0.2276 | Yes |
| 16 | PRC1 |  |  | 242 | 0.830 | 0.2414 | Yes |
| 17 | BIN1 |  |  | 243 | 0.830 | 0.2552 | Yes |
| 18 | TUBA4A |  |  | 246 | 0.827 | 0.2685 | Yes |
| 19 | KNTC1 |  |  | 249 | 0.825 | 0.2817 | Yes |
| 20 | LMNB1 |  |  | 251 | 0.824 | 0.2952 | Yes |
| 21 | VCL |  |  | 278 | 0.778 | 0.3016 | Yes |
| 22 | SMC4 |  |  | 279 | 0.777 | 0.3146 | Yes |
| 23 | CENPE |  |  | 284 | 0.775 | 0.3265 | Yes |
| 24 | KIF4A |  |  | 309 | 0.746 | 0.3329 | Yes |
| 25 | MYH9 |  |  | 323 | 0.735 | 0.3419 | Yes |
| 26 | AURKA |  |  | 360 | 0.705 | 0.3446 | Yes |
| 27 | TTK |  |  | 365 | 0.701 | 0.3553 | Yes |
| 28 | CENPJ |  |  | 376 | 0.695 | 0.3643 | Yes |
| 29 | KIF15 |  |  | 404 | 0.676 | 0.3688 | Yes |
| 30 | SMC3 |  |  | 425 | 0.667 | 0.3749 | Yes |
| 31 | INCENP |  |  | 432 | 0.660 | 0.3844 | Yes |
| 32 | PIF1 |  |  | 445 | 0.653 | 0.3923 | Yes |
| 33 | EZR |  |  | 466 | 0.642 | 0.3980 | Yes |
| 34 | FLNA |  |  | 483 | 0.637 | 0.4046 | Yes |
| 35 | FLNB |  |  | 488 | 0.633 | 0.4141 | Yes |
| 36 | BIRC5 |  |  | 505 | 0.628 | 0.4205 | Yes |
| 37 | ECT2 |  |  | 510 | 0.626 | 0.4300 | Yes |
| 38 | PDLIM5 |  |  | 526 | 0.613 | 0.4364 | Yes |
| 39 | CENPF |  |  | 575 | 0.588 | 0.4342 | Yes |
| 40 | PCNT |  |  | 576 | 0.588 | 0.4440 | Yes |
| 41 | SMC1A |  |  | 618 | 0.570 | 0.4432 | Yes |
| 42 | EPB41L2 |  |  | 623 | 0.569 | 0.4517 | Yes |
| 43 | SASS6 |  |  | 637 | 0.562 | 0.4578 | Yes |
| 44 | NUSAP1 |  |  | 654 | 0.550 | 0.4629 | Yes |
| 45 | ARF6 |  |  | 697 | 0.533 | 0.4613 | Yes |
| 46 | DLGAP5 |  |  | 712 | 0.529 | 0.4666 | Yes |
| 47 | ARHGAP10 |  |  | 744 | 0.518 | 0.4675 | Yes |
| 48 | TUBGCP3 |  |  | 929 | 0.464 | 0.4292 | No |
| 49 | KIF22 |  |  | 975 | 0.450 | 0.4254 | No |
| 50 | MYH10 |  |  | 985 | 0.448 | 0.4306 | No |
| 51 | GEMIN4 |  |  | 1005 | 0.443 | 0.4332 | No |
| 52 | PLK1 |  |  | 1009 | 0.442 | 0.4398 | No |
| 53 | CEP57 |  |  | 1055 | 0.426 | 0.4357 | No |
| 54 | ARHGDIA |  |  | 1093 | 0.419 | 0.4334 | No |
| 55 | NEK2 |  |  | 1100 | 0.417 | 0.4388 | No |
| 56 | BUB1 |  |  | 1145 | 0.408 | 0.4346 | No |
| 57 | CDC42EP1 |  |  | 1193 | 0.398 | 0.4295 | No |
| 58 | ESPL1 |  |  | 1255 | 0.386 | 0.4207 | No |
| 59 | CEP131 |  |  | 1449 | 0.347 | 0.3782 | No |
| 60 | TRIO |  |  | 1493 | 0.342 | 0.3731 | No |
| 61 | CNTROB |  |  | 1528 | 0.335 | 0.3702 | No |
| 62 | CDC27 |  |  | 1562 | 0.330 | 0.3674 | No |
| 63 | RFC1 |  |  | 1579 | 0.328 | 0.3689 | No |
| 64 | SHROOM1 |  |  | 1714 | 0.308 | 0.3405 | No |
| 65 | PALLD |  |  | 1820 | 0.297 | 0.3192 | No |
| 66 | CNTRL |  |  | 1830 | 0.294 | 0.3218 | No |
| 67 | LRPPRC |  |  | 1836 | 0.294 | 0.3255 | No |
| 68 | TBCD |  |  | 1868 | 0.290 | 0.3225 | No |
| 69 | BCAR1 |  |  | 2057 | 0.266 | 0.2799 | No |
| 70 | CEP192 |  |  | 2109 | 0.262 | 0.2715 | No |
| 71 | CKAP5 |  |  | 2135 | 0.258 | 0.2696 | No |
| 72 | CEP72 |  |  | 2137 | 0.258 | 0.2736 | No |
| 73 | DOCK4 |  |  | 2230 | -0.251 | 0.2548 | No |
| 74 | ARFGEF1 |  |  | 2425 | -0.273 | 0.2108 | No |
| 75 | FSCN1 |  |  | 2432 | -0.275 | 0.2139 | No |
| 76 | MID1 |  |  | 2486 | -0.283 | 0.2054 | No |
| 77 | CYTH2 |  |  | 2531 | -0.291 | 0.1992 | No |
| 78 | STK38L |  |  | 2587 | -0.299 | 0.1904 | No |
| 79 | ABI1 |  |  | 2598 | -0.301 | 0.1929 | No |
| 80 | KIF3B |  |  | 2605 | -0.301 | 0.1964 | No |
| 81 | NIN |  |  | 2630 | -0.305 | 0.1955 | No |
| 82 | PCM1 |  |  | 2715 | -0.320 | 0.1798 | No |
| 83 | KLC1 |  |  | 2739 | -0.324 | 0.1795 | No |
| 84 | SHROOM2 |  |  | 2799 | -0.335 | 0.1703 | No |
| 85 | SOS1 |  |  | 2956 | -0.365 | 0.1374 | No |
| 86 | PPP4R2 |  |  | 2989 | -0.370 | 0.1355 | No |
| 87 | FARP1 |  |  | 3001 | -0.373 | 0.1390 | No |
| 88 | ARHGEF12 |  |  | 3022 | -0.378 | 0.1403 | No |
| 89 | CLIP2 |  |  | 3069 | -0.390 | 0.1352 | No |
| 90 | KIF3C |  |  | 3087 | -0.394 | 0.1376 | No |
| 91 | NCK1 |  |  | 3156 | -0.415 | 0.1274 | No |
| 92 | ARL8A |  |  | 3185 | -0.425 | 0.1275 | No |
| 93 | KIFAP3 |  |  | 3312 | -0.461 | 0.1037 | No |
| 94 | RABGAP1 |  |  | 3347 | -0.475 | 0.1031 | No |
| 95 | MARCKS |  |  | 3369 | -0.487 | 0.1059 | No |
| 96 | APC |  |  | 3422 | -0.510 | 0.1014 | No |
| 97 | GSN |  |  | 3487 | -0.537 | 0.0943 | No |
| 98 | SPTBN1 |  |  | 3515 | -0.551 | 0.0967 | No |
| 99 | PREX1 |  |  | 3716 | -0.698 | 0.0583 | No |
| 100 | CCDC88A |  |  | 3854 | -0.848 | 0.0382 | No |
| 101 | FGD4 |  |  | 4014 | -1.358 | 0.0210 | No |
Table: GSEA details [plain text format]

  

Fig 2: HALLMARK\_MITOTIC\_SPINDLE: Random ES distribution      
 Gene set null distribution of ES for **HALLMARK\_MITOTIC\_SPINDLE**
